# Identification and Functional Characterization of a Novel Immunomodulatory Protein From *Morchella conica* SH

**DOI:** 10.3389/fimmu.2020.559770

**Published:** 2020-10-26

**Authors:** Guogan Wu, Yu Sun, Tingshan Deng, Lili Song, Peng Li, Haijuan Zeng, Xueming Tang

**Affiliations:** Biotechnology Research Institute, Shanghai Academy of Agricultural Sciences, Shanghai, China

**Keywords:** fungal immunomodulatory protein, FIP-mco, rFIP-mco, *Morchella conica* SH, *Pichia pastoris* X33, anti-tumor, immunomodulatory

## Abstract

A novel fungal immunomodulatory protein (FIP) was found in the precious medical and edible mushroom *Morchella conica* SH, defined as FIP-mco, which belongs to the FIP family. Phylogenetic analyses of FIPs from different origins were performed using Neighbor-Joining method. It was found that FIP-mco belonged to a new branch of the FIP family and may evolved from a different ancestor compared with most other FIPs. The cDNA sequence of FIP-mco was cloned and expressed in the yeast *Pichia Pastoris* X33. The recombinant protein of FIP-mco (rFIP-mco) was purified by agarose Ni chromatography and determined by sodium dodecyl sulfate-polyacrylamide gel electrophoresis (SDS-PAGE) and Western blot analysis. The protein rFIP-mco could significantly suppress the proliferation of A549 and HepG2 cells at the concentration of 15 and 5 μg/ml, respectively, and inhibited the migration and invasion of human A549 and HepG2 cells at the concentration of 15 and 30 μg/ml respectively *in vitro*. Further, rFIP-mco can significantly reduce the expression levels of TNF-α, IL-1β, and IL-6 in the THP1 cells (human myeloid leukemia mononuclear cells). In order to explore the potential mechanism of the cytotoxicity effect of rFIP-mco on A549 and HepG2 cells, cell cycle and apoptosis assay in the two cancer cells were conducted. The results demonstrated that G0/G1 to S-phase arrest and increased apoptosis may contribute to the proliferation inhibition by rFIP-mco in the two cancer cells. Molecular mechanism of rFIP-mco’s reduction effect on the inflammatory cytokines was also studied by suppression of the NF-κB signaling pathway. It showed that suppression of NF-κB signaling is responsible for the reduction of inflammatory cytokines by rFIP-mco. The results indicated the prospect of FIP-mco from *M. conica* SH as an effective and feasible source for cancer therapeutic studies and medical applications.

## Introduction

Edible mushrooms are famous for their highly nutrient constitution, as well as their tonic and medicinal values in medical applications. In recent decades, medicinal mushrooms has aroused much attention for their antitumor, immunomodulating, antiviral, hypocholesterolemia, and hepatoprotective activities (1). Many bioactive components isolated from edible mushrooms have been studied, including polysaccharides, terpenoids, lectins, proteins, and organic compounds (1, 2). They have been isolated not only from fruiting body but also the mycelium, and possess various chemical characteristics and biological effects on immune cells (2, 3).

*Morels* (*Morchella* spp.) are precious rare edible fungi famous for their delicious taste and rich nutrients. True morels are ascomycetous fungi with high reputation for their edibility and appearance, which is similar to “a sponge on a stick” (4, 5). Due to their unique flavor and nutritional value, these morels have been used in soups and gravies, as a source of medicinal adaptogens, immunostimulants, and potential anti-tumor agents (6–8). It is also widely used in traditional Chinese medicine to treat indigestion, excessive phlegm and shortness of breath (9, 10).

*Morchella conica* is an edible mushroom belonging to the genus *Morchella*. It prefers humid areas covered by deciduous and coniferous forests subjected to seasonal flooding (11, 12). It has been consumed as tasted food. Despite the popularity of morels, the difficulties of culturing wild *M. conica* make them very expensive in food markets (13). On its molecular study, several components isolated from *M. conica*, including polysaccharides, have been reported to exhibit immunomodulatory, antitumor, and antioxidant activities. For example, a kind of polysaccharide NMCP-2 from *M. conica Pers*. shows antioxidant activities against H_2_O_2_ in human embryonic kidney cells (14). Another two kinds of polysaccharides from *M. conica* demonstrate their inhibition on nitric oxide production in macrophages (2). However, numerous aspects of their biological features such as life cycle and ecology remain poorly understood, especially little is known on molecular study of the species.

In recent decades, an immunomodulatory component from mushrooms, fungal immunomodulatory protein (FIP), has been a new area due to its potential applications in anti-tumor, anti-anaphylactic, and immunomodulatory activities (15). FIPs are a type of small protein (110–114 amino acids, approximately 13 KD) which possesses immunomodulatory function. FIP-fve was first reported in *Flammulina velutipes*. At present, there are at least twenty FIPs been reported (**Supplementary Table S1**) (16–41). FIPs lack His, Cys and Met residues but are rich in Asp and Val residues (6). They share a Fve conserved domain (PF09259). FIPs are considered as a T cell activator which can stimulate several important cytokines and molecular factors (IL-2, IL-3, IFN-γ, and TNF-α etc.), and can inhibit progression or migration of several kinds of cancer cells (15, 42). However, homologous FIPs from different sources could display diverse activities and anti-tumor mechanisms, for example, FIP-gts from *Ganoderma tsugae* induces premature senescence of A549 lung cancer cells, while LZ-8 from *Ganoderma lucidum* increases A549 G1 arrest, and FIP-sch3 (FIP from *Stachybotrys chartarum*) induces A549 apoptosis and inhibits migration (36, 43, 44).

To our best knowledge, until now, ten FIPs have been extracted from fruit body or mycelium: LZ-8, FIP-fve, FIP-vvo, FIP-gts, ACA, APP, PCP, FIP-her, FIP-gts, and YZP. Eleven FIPs have been identified using homologous cloning method: FIP-cve, FIP-gap1, FIP-gap2, FIP-gat, FIP-gja, FIP-gmi, FIP-gsi, FIP- tvc, FIP-cru, FIP-gat, and LZ-9. As the development of Next Generation Sequencing, genome mining has been applied as a new tool for searching potential FIPs. FIP-nha, FIP-bbo, FIP-sch2, FIP-sch3, FIP-lrh, FIP-dsq2, c13717, FIP-vv082, and FIP-ppl were cloned directly by genome mining.

The low yield of native FIPs extracted from mushrooms is a major limitation in the application of FIPs, and there’s neither report about the native FIP nor its recombinant expression and immunomodulatory studies in *M. conica*. Therefore, in this paper, FIP-mco, was first obtained from the precious rare edible fungus *M. conica* SH sequenced by our lab. The *P. pastoris* expression system was employed to successfully express the recombinant protein of FIP-mco (rFIP-mco). The phylogenetic analysis of FIP-mco indicated that FIP-mco is evolutionarily more closed to YZP, ACA, and APP. They share a relatively conserved amino acid sequence and functional similarity, suggesting that this cluster of FIPs could be characterized as a novel immunomodulatory family. The protein rFIP-mco could suppress the proliferation of human A549 and HepG2 cancer cells significantly at the concentration of 15 and 5 μg/ml, and could remarkably inhibit the migration of A549 and HepG2 cancer cells at the concentration of 15 and 30 μg/ml respectively *in vitro*. Through cell cycle and apoptosis assay in A549 and HepG2 cancer cells, it was found that G0/G1 to S-phase arrest and increased apoptosis may contribute to the proliferation inhibition by rFIP-mco in the two cancer cells. Further, rFIP-mco could significantly reduce the expression levels of inflammatory cytokines, TNF-α, IL-1β, and IL-6 by suppressing the NF-κB signaling pathway. The immunomodulatory and anti-tumor activities will surely render *M. conica* SH high pharmaceutical and economical values.

## Materials and Methods

### Strains and Media

*M. conica* SH was collected from a local farm affiliated to Yunnan Academy of Agricultural Sciences, Yunnan Province, China. *M. conica* SH was sequenced with a whole-genome shotgun sequencing strategy. The sequencing data was submitted to NCBI database (NCBI Sequencing Program VOVZ00000000). Yeast extract, peptone, macro agar, yeast nitrogen base (YNB) with ammonium sulfate without amino acids, biotin, buffered glycerol-complex medium (BMGY), glycerol and methanol were purchased from Sangon (Shanghai, China). Selective marker zeocin was purchased from Invitrogen (California, USA). LB agar medium supplemented with kanamycin was used for *E. coli* DH10B cultivation, yeast extract peptone dextrose (YPD) supplemented with zeocin was used for *P. pastoris* X33 cultivation, BMGY was used for the recombinant FIP-mco expression.

### Data and Bioinformatics Analysis

Amino acid sequences of FIPs listed in **Supplementary Table S1** were downloaded from NCBI. Reference sequences were used as queries for BLASTp searches (http://www.ncbi.nlm.njh.gov/BLAST/) to identify homologs in the seven examined edible mushroom, *A. bisporus* (45), *L. edodes* (46), *L. tigrinus* (47), *M. importuna* (48), *C. cinerea* (49), *P. ostreatus* (50), and *M. conica* SH. Putative orthologues were verified by sequence alignment using MUSCLE (https://www.ebi.ac.uk/Tools/msa/muscle/) (51). The secondary structures of FIPs were predicted using the PSIPRED program (http://bioinf.cs.ucl.ac.uk/psipred/) (52). Phylogenetic analyses using Neighbor-Joining (NJ) were performed by MEGA7 (https://megasoftware.net) (53) with Kimura 2-parameter model and statistical bootstrapping procedure involving 1,000 replicates.

### Construction of the Recombinant Plasmid pPICZαA-fipmco

Local BLASTp (http://www.ncbi.nlm.njh.gov/BLAST/) using reported FIP sequences (**Supplementary Table S1**) against *M. conica* SH genome was performed, a putative FIP protein (scaffold3t326) was obtained. The putative FIP protein shared 50% identity with the reported immunomodulatory protein ACA (16), and this candidate was designated as FIP-mco. According to the *M. conica* SH genome information, the coding sequence (CDS) of FIP-mco was identified and optimized for *P. pastoris* X33 codon usage preference, the putative CDS of FIP-mco was subsequently synthesized (Sangon, Shanghai, China) with restriction enzyme site *EoR*I at the 5’ end and *Not*I at the 3’ end. Synthesized FIP-mco CDS was digested and cloned into the corresponding restriction enzyme sites in pPICZαA expression vector (Invitrogen, California, USA), resulting in the recombinant plasmid pPICZαA-fipmco. The recombinant plasmid pPICZαA-fipmco was transformed into *E. coli* DH10B, and the positive colonies were isolated and confirmed by sequencing with 5’/3’ AOX primers. The verified pPICZα-fipmco was linearized by SacI digestion. Transformation of the linearized DNA was performed according to the pPICZαA manual by Invitrogen. Transformed cells were spread onto YPD plates supplemented with 100 ìg/mL Zeocin, the positive colonies were isolated, and were confirmed for integration by PCR with 5’/3’ AOX primers. The correctly integrated isolates were used for protein expression and purification.

### Expression and Purification of rFIP-mco

The correct integrant colony was grown in 20 ml of BMGY medium at 30°C, 220 rpm for 24 h. The yeast cells were collected by centrifugation at 1,500×*g* for 5 min at 4°C, and subsequently resuspended in 750-ml BMMY medium (flask, 220 rpm) to a final OD_600_ of 1.0. The expression of rFIP-mco was induced by adding 100% methanol to a final concentration of 0.5% every 12 h for 72 h. The secreted rFIP-mco was collected and purified *via* His-affinity Ni-NTA chromatography (Qiagen, California, USA) according to the manufacturer’s protocols, and was dialyzed third times against PBS in the total period of 18 h. The total rFIP-mco protein mass was detected using BCA kit (Sangon, Shanghai, China), whereas the protein mass of the purified rFIP-mco was adjusted with extinction coefficient predicted according to the amino acid sequence (54).

### SDS-PAGE and Western Blotting

FIP-mco was examined by SDS-PAGE and Western blot analysis. For SDS-PAGE, 20-µl supernatant of the culture transformed with pPICZαA-fipmco was mixed with 6 × loading buffer, boiled and centrifuged before electrophoresis. The sample was then loaded onto 12% resolving acrylamide gel. After 120 V, 60-min electrophoresis in the separation gel and 80 V, 20 min in the concentrated gel, the gel was subjected to staining with Coomassie brilliant blue G250. The supernatant of *P. pastoris* X33 without the recombinant plasmid was used as control. For Western blotting, it was performed according to the manufacturer’s protocols (Bio-Rad, California, USA). The protein resolved in SDS-PAGE was transferred to a PVDF membrane. After blocked by 5% nonfat dry milk and washed using TBST for three times, the membrane was incubated with 1:500 diluted anti-6×His rabbit polyclonal antibody (Concentration: 0.6 mg/ml, D110002, Sangon, Shanghai, China) as the primary antibody with gentle agitation at 37°C for 1 h, and then incubated with 1:8,000 HPR-conjugated goat anti-rabbit IgG (Concentration: 0.2 mg/ml, D110058, Sangon, Shanghai, China) as the secondary antibody at 37°C for 1 h while gently agitated, finally stained with TMB (TMB color reagent B solution).

### Cell Lines and Cell Culture

Cell lines (A549, HepG2, and THP-1) were obtained from the Cell Bank of Type Culture Collection of the Chinese Academy of Sciences, Shanghai Institute of Cell Biology (Shanghai, China). Cells were grown in DMEM (Thermo, NH, USA) containing 10% FBS (Thermo, NH, USA). In addition, all cell media was supplemented with penicillin (100 U/ml) and streptomycin (100 U/ml) at 37°C in a 5% CO_2_ incubator.

### Cytotoxicity Assay in A549 and HepG2 Cancer Cells

The Cell-Counting Kit 8 (CCK-8) was utilized to evaluate the viability of A549 and HepG2 cells according to the manufacturer’s protocols (Dojindo Molecular Technologies, Japan). Twenty-four hours after seeding cells in 96-well plates, different concentrations of rFIP-mco (5, 15, 30 μg/ml) were delivered for 1, 2, 3, and 4 days. Complete medium (100 µl) along with CCK-8 solution (10 µl) was used to replace the medium in each well followed by a 60-min incubation at 37°C. A Multiskan Spectrum (Thermo Fisher, Rockford, IL, USA) was utilized to measure the absorbance at 450 nm.

### Migration and Invasion Inhibition Assay of A549 and HepG2 Cancer Cells

Cell migration was assessed using wound healing assay. The cells were grown until 80-90% confluent in 6-well plates, at which time a 10-ml sterile pipette tip was used to create a scratch wound in the cell monolayer. Media containing 2% FBS was then added to the cells for 24 h, after which a phase-contrast microscope (OLYMPUS CKX53) was used to image cells. To assess cell invasion, 8 μm pore size inserts were used for invasion assay with the upper surface of the membrane being coated using Matrigel (BD Biosciences, CA, USA) based on provided directions. The protein rFIP-mco was added into A549 and HepG2 cells at the concentration of 0, 5, 15, and 30 μg/ml, respectively. After coating, 2 × 10^5^ cells were added to the upper chamber for 24 h at 37°C, and then those cells that had not invaded were removed using a cotton swab. Methanol was used to fix invasive cells at the bottom of the Matrigel, followed by leucocrystal violet staining and counting of cells *via* microscopy.

### Inflammatory Cytokine Induction by rFIP-mco

To evaluate the immunomodulatory bioactivities of rFIP-mco, the levels of induced TNF-α, IL-1β, and IL-6 released from the THP-1 cells were detected. The THP-1 cells were prepared, recovered and cultured in 6-well plates with 5 × 10^5^ cells per well in 5% CO_2_ at 37°C for 24 h. RFIP-mco was added into the THP-1 cell culture which was pretreated with 1 μg/ml lipopolysaccharide (LPS) at the concentration of 0, 5, 15, and 30 μg/ml, respectively. After 24 h of incubation, the levels of TNF-α, IL-1β, and IL-6 in the supernatants were detected using ELISA Kits according to the manufacturer’s instructions (R&D, Minnesota, USA). Ultimately, the absorbance was determined with a microplate reader (Model 680, Bio-Rad, Hercules, CA, USA). Human IL-6 Quantikine ELISA Kit (R&D, D6050), Human IL-1β Quantikine ELISA Kit (R&D, DLB50), Human TNF-α Quantikine ELISA Kit were all purchased from Dakewe. The THP-1 cell culture without adding rFIP-mco and cells added with 30 μg/ml rFIP-mco were used as control.

### Flow Cytometry Analysis of Cell Cycle

A549 and HepG2 cells were harvested after treatment with rFIP-mco at the concentration of 0, 5, 15, and 30 μg/ml for 48 h, and phosphate-buffered saline (PBS) washing was performed followed by an overnight fixation at 4°C in 75% ethanol. RNase A (Sigma-Aldrich) was added for 30 min at 37°C to remove RNA. Propidium iodide (PI) solution (Sigma-Aldrich) was added to the fixed cells, incubated at room temperature for 30 min, and assayed using a FACS Aria I flow cytometer (BD Biosciences).

### Flow Cytometry Analysis of Apoptosis

Apoptosis assay was performed using FITC Annexin V Apoptosis Detection Kit I (BD Biosciences). Briefly, A549 and HepG2 cells were harvested using trypsin after treatment with rFIP-mco at the concentration of 0, 5, 15, and 30 μg/ml for 48 h, washed twice with ice-cold phosphate-buffered saline (PBS) and resuspended in 1 × Binding Buffer at a concentration of 1 × 106 cells/ml. Solution (100 μl) was transferred into a 5-ml culture tube, and then 5-μl PI and 5-μl FITC Annexin V were added. After incubated for 15 min at 25°C in the dark, 400-μl 1×Binding Buffer was added to each tube and stained cells were analyzed by FACS Calibur Flow Cytometer (BD Biosciences).

### Western Blot Analysis for NF-κB Pathway

Cells were added into cytoplasmic and nuclear extracts by NE-PER ® Nuclear and Cytoplasmic Extraction Reagents. Protein concentrations of the extracts were measured by BCA assay. Rabbit anti-IκBα antibody was purchased from Cell Signaling Technology. Rabbit anti-β-Actin antibody and anti-Histone 3 antibody were purchased from Abcam (Catalog number ab8227, ab1791). Mouse anti-NF**-**κB p65 subunit antibody was purchased from Santa Cruz. Western blot analyses were performed as previously described (55). Actin level in cytoplasmic extraction and Histone 3 in nuclear extraction were detected to show equal protein loading.

### Luciferase Reporter Gene Assay

The pRL-TK and NF-κB reporter plasmid pGL4.32, and dual luciferase reporter assay system were all purchased from Promega. THP-1 cells were seeded into 96-well plates, and co-transfected with NF-κB reporter plasmid (80 ng/well) and pRL-TK (8 ng/well) as an internal control. After 12 h of incubation, the cells were serum deprived for 12 h and subjected to LPS stimulation with or without rFIP-mco. The cell lysates were prepared with passive lysis buffer, and the luciferase activity was measured by Luciferase Reporter Assay System (Promega) and normalized by the transfection efficiency calculated by the Renilla luciferase activity from pRL-TK.

### Statistical Data Analysis and Software

All experimental data are presented as the mean ± SD from at least three independent tests performed in triplicate (n = 3). ANOVA was used for statistical data analysis, and Graphpad was used to analyze in the cell experiment. Differences were deemed to be statistically significant at *p* < 0.05.

## Results

### Sequence Mining and Analysis of FIP-mco and FIPs From Different Origins

Local BLAST using reported FIPs (**Supplementary Table S1**) against *M. conica* SH genome (NCBI Sequencing program VOVZ00000000) revealed one predicted protein (scaffold3t326) with significant sequence similarity to ACA (percent identity: 50%) (16) and YZP (percent identity: 41.6%) (39). This predicted protein, FIP-mco, consisted of 133 amino acid residues with a calculated molecular weight of 13.57 kDa and an isoelectric point (pI) of 4.53. The amino acid composition of FIP-mco lacks His and Met but is rich in Ala, Thr, and Val residues, which is similar to the typical FIP sequence characteristics (6).

**Figure 1:**
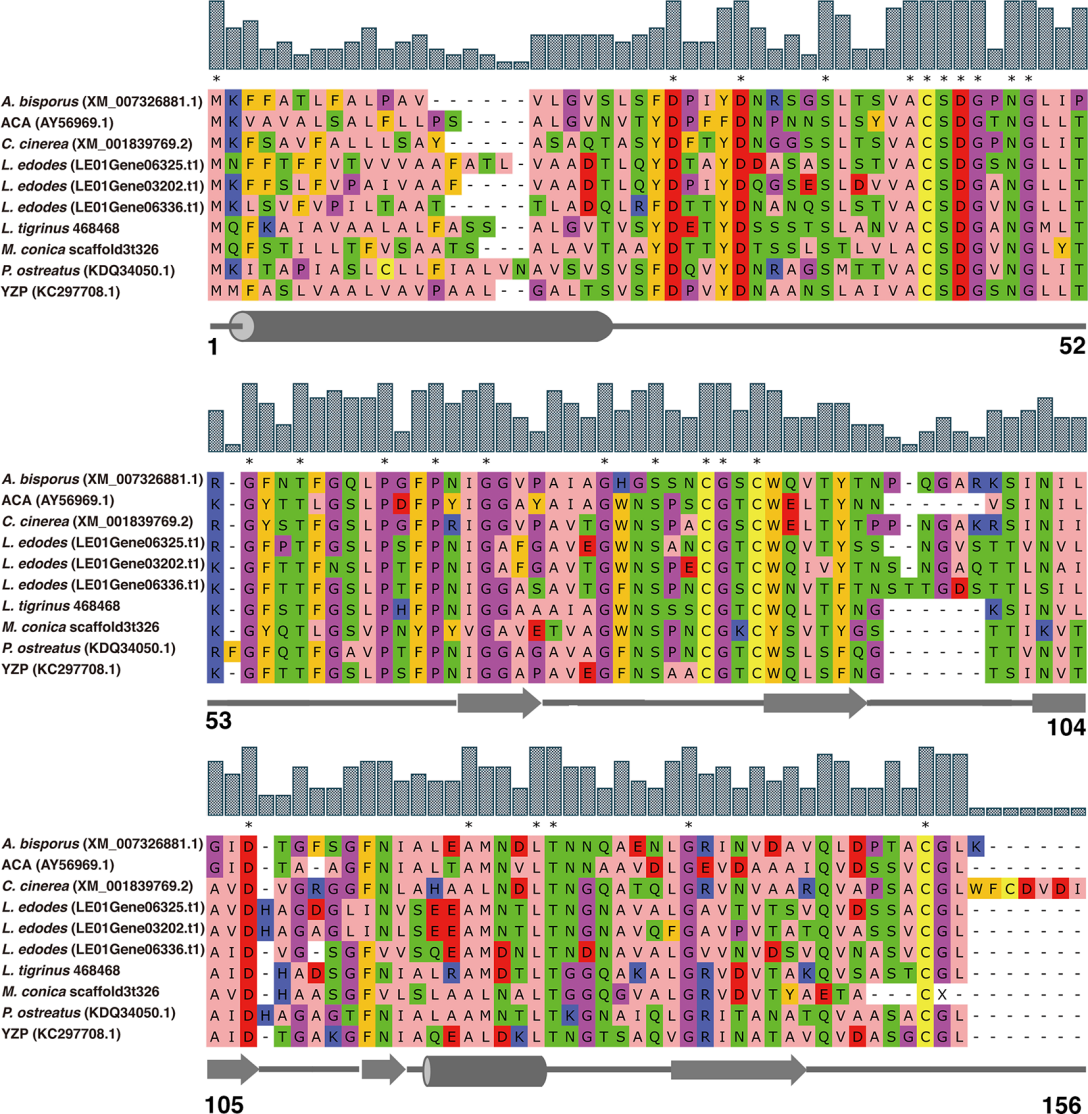
Amino acid sequence alignment of FIPs with cerato-platanin motif. The alignment was performed by MUSCLE. ACA and YZP were from *A. camphorata* and *T. versicolor*, respectively. Other FIP-like proteins, *A. bisporus*, *L. edodes*, *L. tigrinus*, *C. cinerea*, *P. ostreatus*, and *M. conica* SH, were selected by genome mining using the sequences of ACA and YZP as baits. NCBI accession numbers are addressed in brackets. Amino acids residues are colored according to their physicochemical properties, alphatic/hydrophobic (residues ILVAM) - rose; aromatic (FWY) - orange; positive (KRH) - dark blue; negative (DE) - red; hydrophilic (STNQ) - light green; conformationally special (PG) - magenta; cysteine (C) - yellow. Bar above amino acids residues represent consensus level, and consensus amino acid residues were labeled by asterisk. Secondary structure predictions are shown under the sequences, cylinders represent α helices, arrows represent β sheets, and straight lines represent random coils. Amino acid residue positions are numbered in last row.

**Figure 2:**
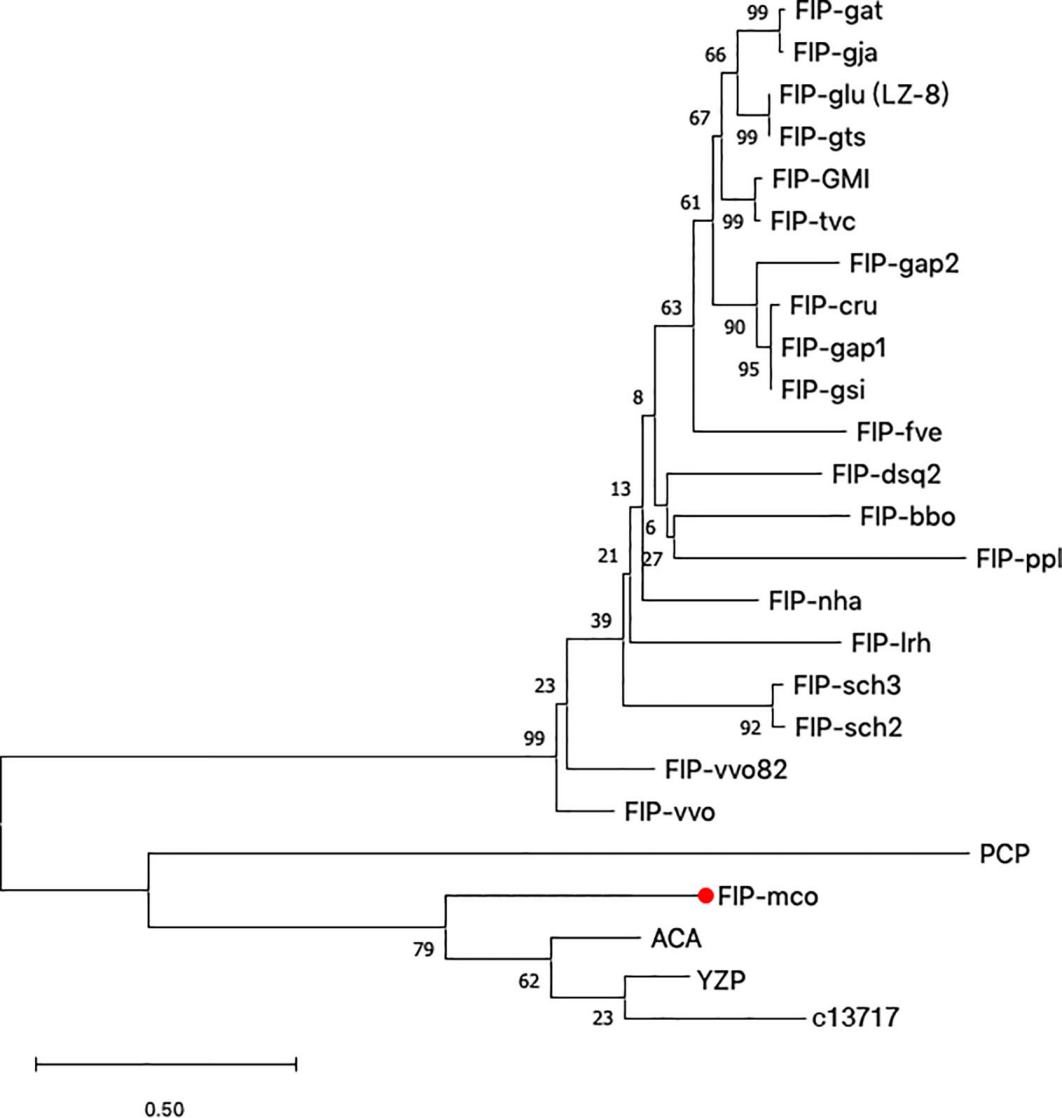
Phylogenetic tree based on protein sequences of selected FIPs. 24 amino acid sequences (**Supplementary Table S1**) were analyzed. The evolutionary relationship between FIPs were inferred using the Neighbor-Joining method, and Jones-Taylor-Thornton (JTT) model was used to compute evolutionary distances. The numbers at the nodes indicate bootstrap support values (%). The scale bar represents 0.5 substitutions per site.

Along with *M. conica* SH, we chose the other six reported genomes: *A. bisporus* (45), *L. edodes* (46), *L. tigrinus* (47), *M. importuna* (48), *C. cinerea* (49), and *P. ostreatus* (50) for further bioinformatics analysis. It revealed five homologs in the genomes by BLASTp using the amino acid sequences of ACA and YZP (**Figure 1**) (16, 39, 45–47, 49–51). Secondary structure prediction of FIP-mco and its homologs showed high structural similarity between the seven predicted proteins (**Figure 1**). All of the proteins consist of two α helices and seven β sheets, indicating a similar folding pattern which could give insight into similar structural motifs or potential function between the proteins. Through the construction of the phylogenetic tree, it showed that FIPs from *Ganoderma* were clustered into one lineage, indicating that they are highly conserved among the genus. Interestingly, FIP-tvc and FIP-cru were also clustered in the lineage. FIP-dsq2, FIP-bbo, and FIP-ppl were clustering in a separate lineage. Above FIPs were included in a primary lineage compared with other relatively isolated FIPs, such as FIP-nha and FIP-lrh. FIP-mco, PCP, ACA, and YZP were clustered into another lineage, and there was substantial evolutionary distance between them and the FIPs mentioned above. The results indicated that FIPs were likely derived from two separate ancestors (**Figure 2**).

### Identification of rFIP-mco From *M. conica* SH

**Figure 3:**
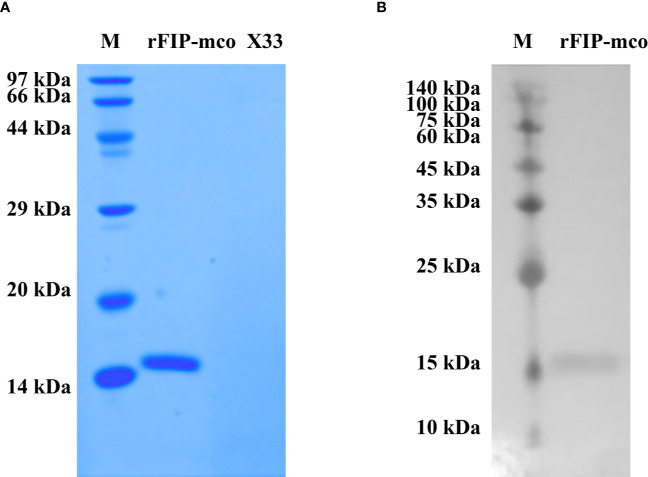
Expression and identification of rFIP-mco. **(A)** SDS-PAGE of rFIP-mco; supernatant of X33 strain broth as control (X33); Lane M - marker. **(B)** Western blot analysis of rFIP-mco; Lane M - marker.

By BLAST, a new FIP candidate protein was found in *M. conica* SH. FIP-mco encoding gene was obtained and synthesized from the sequenced *M. conica* SH, designated FIP-mco. FIP-mco comprised 399 bp with 133 amino acids. After expression in *Pichia pastoris* X33 and subsequent purification, the protein sample was subjected to SDS-PAGE and Western blot analysis. A single band of rFIP-mco was observed near 13 kDa in SDS-PAGE (**Figure 3A**), which was approximately the calculated theoretical size of the protein. Western blot using 6 × His-tag antibody suggested the recombinant protein had been expressed and possessed the immune recognition (**Figure 3B**). By determination of the SDS-PAGE and Western blot, the recombinant protein of FIP-mco (rFIP-mco) was successfully expressed, purified, and identified.

### Inhibitory Effect of rFIP-mco on the Proliferation of A549 and HepG2 Cells

**Figure 4:**
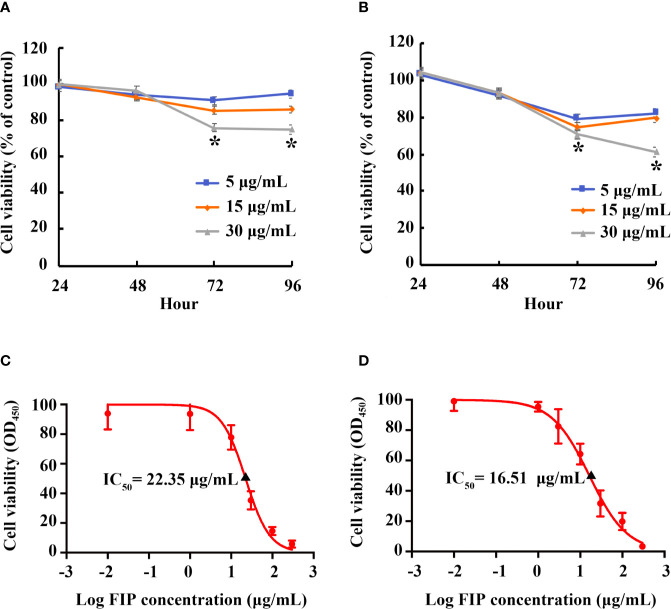
Effects of rFIP-mco on A549 and HepG2 cell proliferation. A549 **(A)** or HepG2 **(B)** cells treated with gradient concentrations of rFIP-mco. Cell proliferation was determined by a Cell-Counting Kit-8 assay for 0, 24, 48, 72 and 96 h. *P < 0.05 vs. control groups (untreated cells). IC_50_ value of rFIP-mco for A549 **(C)** and HepG2 **(D)** cells were also determined.

By CCK-8 assay, effect of rFIP-mco on A549 and HepG2 cell proliferation was observed at different concentrations (5, 15, 30 μg/ml). The proliferation of the cells could be suppressed at all the set concentrations. At the concentration of 15 μg/ml, rFIP-mco can remarkably inhibit the proliferation of A549 cells (**Figure 4A**). Treatment of A549 cells with 15 μg/ml rFIP-mco for 48 h and 72 h resulted in inhibition by 6.91% and 14.48%, respectively. At the concentration of 5 μg/ml, rFIP-mco can remarkably inhibit the proliferation of HepG2 cells (**Figure 4B**). Treatment of HepG2 cells with 5 μg/ml rFIP-mco for 48 h and 72 h resulted in inhibition by 8.06% and 20.68%, respectively. IC_50_ values of rFIP-mco in A549 and HepG2 cells were also determined, which were 22.35 and 16.51 μg/ml, respectively (**Figures 4C, D**). The results indicated that rFIP-mco could be used as a potent tumor inhibitor in A549 and HepG2 cells, and rFIP-mco showed a stronger effect in HepG2 cells.

### rFIP-mco Inhibits the Migration and Invasion of A549 and HepG2 cells

**Figure 5:**
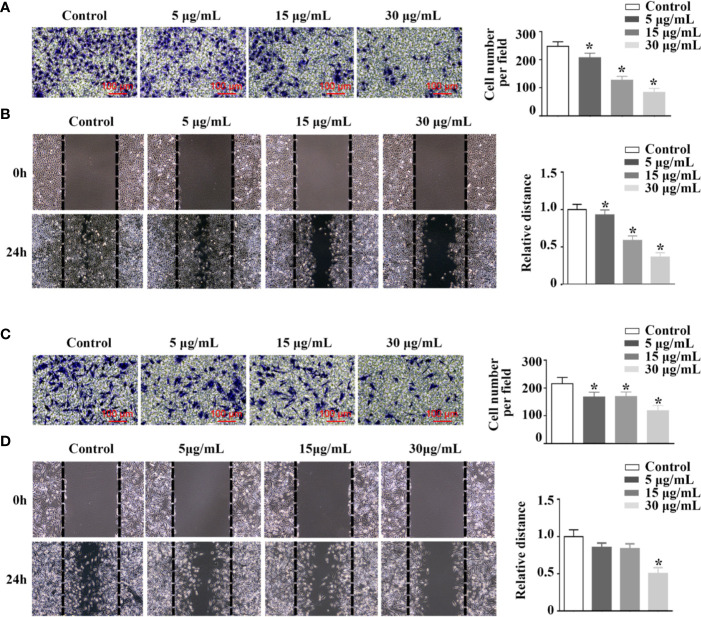
Effects of rFIP-mco on cell migration and invasion. Transwell migration assay **(A)** and cell migration monitored by the wound healing assay **(B)** were conducted in A549 cells treated with different concentrations of rFIP-mco. Transwell migration assay **(C)** and cell migration monitored by the wound healing assay **(D)** were carried out in HepG2 cells treated with different concentrations of rFIP-mco. *P < 0.05 vs. control groups (untreated cells).

By transwell and wound healing assay, effect of rFIP-mco on A549 and HepG2 cell migration and invasion was observed. At the concentration of 15 μg/ml, rFIP-mco could significantly inhibit the migration and invasion of A549 cells (**Figures 5A, B**). At the concentration of 30 μg/ml, rFIP-mco could remarkably inhibit the migration and invasion of HepG2 cells (**Figures 5C, D**). It showed that rFIP-mco could both inhibit the migration and invasion of the two cancer cells, indicating the application prospect of rFIP-mco as antitumor therapeutic adjuvant. In the test of rFIP-mco’s effect on migration and invasion, the result was different to the proliferation inhibit effect of rFIP-mco on A549 and HepG2 cells, in which HepG2 cells were more sensitive to rFIP-mco. The migration and invasion of A549 cells were inhibited by rFIP-mco at a relative low concentration compared with HepG2 cells. It displayed a more potent inhibition effect in A549 cancer cells in the migration and invasion assay.

### The Effect of rFIP-mco on the Cell Cycle and Apoptosis in A549 and HepG2 Cells

**Figure 6:**
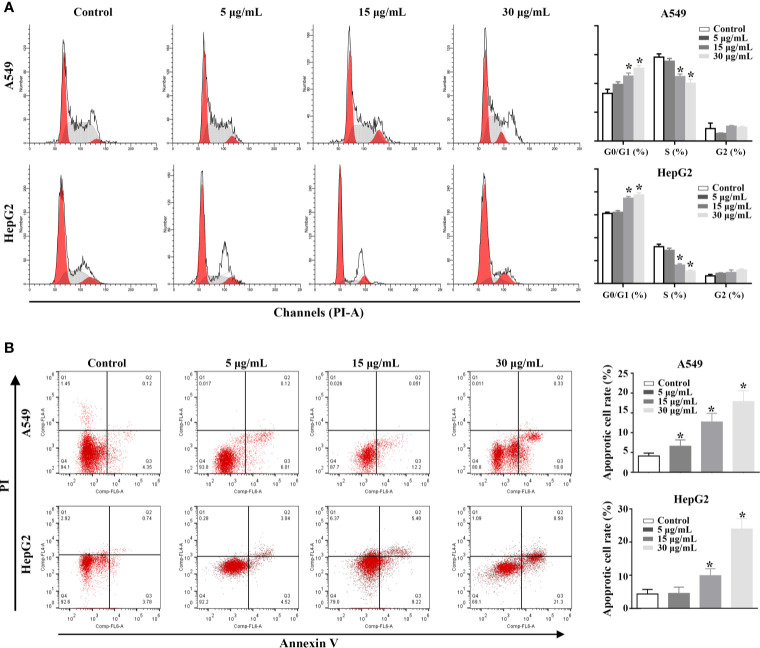
Effects of rFIP-mco on cell cycle and apoptosis in A549 and HepG2 cells were determined using flowing cytometry analysis. **(A)** The effect of rFIP-mco on cell cycle of A549 and HepG2 cells. **(B)** The effect of rFIP-mco on apoptosis in A549 and HepG2 cells. *P < 0.05.

In order to explore the potential mechanism of the proliferation inhibitory effect of rFIP-mco on A549 and HepG2 cells, cell cycle, and apoptosis assay in the two cancer cells were conducted. The analysis of cell-cycle distribution revealed that rFIP-mco significantly decreased the percentage of S-phase cells and increased the percentage of G0/G1-phase cells, indicating that the increase amount of rFIP-mco caused a G0/G1 arrest in A549 and HepG2 cells (**Figure 6A**). Annexin V staining showed that the percentage of early apoptotic cells following rFIP-mco treatment was drastically increased compared with that in control groups (**Figure 6B**), indicating that the addition of rFIP-mco induced cell apoptosis of A549 and HepG2 cells. The effect of rFIP-mco on cell apoptosis was more significant in HepG2 cells than that in A549 cells. These data imply that G0/G1 to S-phase arrest and increased apoptosis may contribute to the proliferation inhibition by rFIP-mco in the two cancer cells.

### rFIP-mco Reduces the Expression Level of TNF-α, IL-1β, and IL-6

**Figure 7:**
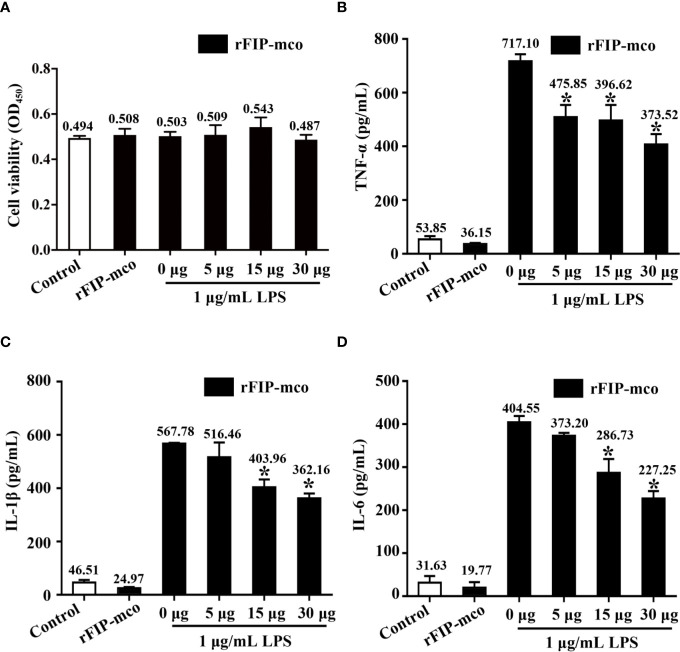
Effects of rFIP-mco on inflammatory cytokines in LPS treated THP-1 cells. Cells were stimulated with LPS (1 μg/ml) for 24 h, and then were treated with gradient concentration of rFIP-mco for 24 h. **(A)** Cell viability of the THP-1 cells were determined. **(B)** TNF-α, **(C)** IL-1β, and **(D)** IL-6 releases were analyzed by ELISA. *P < 0.05 vs. control groups (untreated cells).

Three cytokines, TNF-α, IL-1β, and IL-6 were detected to evaluate the immunomodulatory activity of rFIP-mco. The results showed that the cell viability of the THP1 cells were almost not affected by rFIP-mco at different concentrations (**Figure 7A**). By the ELISA assay, it showed that rFIP-mco could significantly reduce the levels of TNF-α, IL-1β, and IL-6 (**Figures 7B–D**). The protein rFIP-mco (30 μg/ml) reduced the expression level of TNF-α from 717.10 to 373.52 pg/ml. The expression level of IL-1β was reduced from 567.78 to 362.16 pg/ml when added with 30 μg/ml rFIP-mco. For IL-6, the value was 404.55 to 227.25 pg/ml. In the three inflammatory cytokines, rFIP-mco had a more significant effect on TNF-α than on IL-1β and IL-6. In this experiment, the immunomodulatory protein rFIP-mco had no influence on the cell viability of the THP-1 cells but reduced the expression level of the three cytokines, TNF-α, IL-1β, and IL-6 significantly in THP-1 cells.

### rFIP-mco Reduces the Release of Inflammatory Cytokines by Blocking the NF-κB Pathway

**Figure 8:**
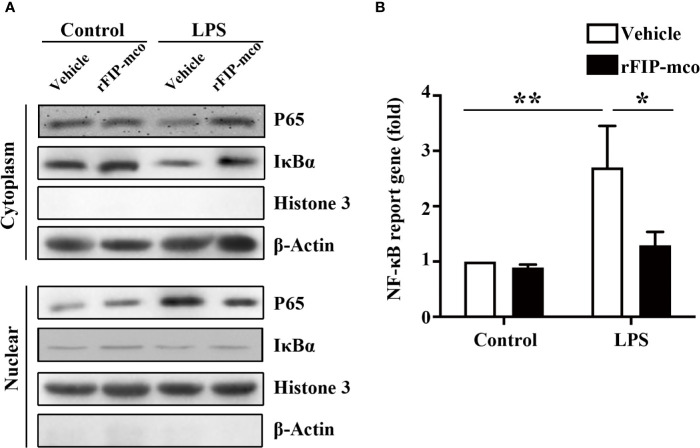
Western blot analysis for NF-κB pathway and Luciferase reporter gene assay. The relative nuclear and cytoplasmic level of NF-κB p65 subunit and IκBα were determined by Western blot analysis by treatment with 30 μg/ml rFIP-mco **(A)**. Luciferase reporter gene assay was conducted to examine the activation of NF-κB signaling pathway by treatment with 30 μg/ml rFIP-mco **(B)**. *P < 0.05 vs. control groups, **P < 0.01 vs. control groups.

To determine the molecular mechanism of the reduced expression level of TNF-α, IL-1β, and IL-6 induced by rFIP-mco, the relative nuclear level of NF-κB p65 subunit and the cytoplasmic level of IκBα were determined by Western blot analysis. The nucleic p65 level was significantly enhanced, while IκBα was degraded apparently in the cytoplasm of THP1 cells. The increase of p65 expression and IκBα degradation was remarkedly blocked by the treatment with 30 μg/ml rFIP-mco (**Figure 8A**). The results of luciferase reporter gene assay revealed that rFIP-mco could significantly inhibit the NF-κB-luciferase reporter gene activity in THP1 cells (**Figure 8B**). It demonstrated that suppression of NF-κB signaling was responsible for the reduction of inflammatory cytokines by rFIP-mco.

## Discussion

At present, more than twenty FIPs have been isolated or synthesized since the first FIP, LingZhi-8 (LZ-8 or FIP-glu), was isolated in 1989 (26). The FIPs from different origins not only share high homology in nucleotides and amino acid sequences, but also show significant similarity in immunomodulation and anti-tumor activities (6, 42).

BLAST results suggested that more than sixteen FIPs share a Fve conserved domain. The Fve protein family showed significant immunomodulatory activity associated with many biologic processes (15, 42). Noticeably, there are an exception in the reported FIPs, ACA, YZP, and c13717 (**Supplementary Table S1**), which share low identities (<10%) to those FIPs from the Fve family. They share a conserved cerato-platanin (pfam07249) domain. Proteins from this family were usually involved in host-pathogen interaction and induction of cell necrosis and phytoalexin synthesis which are part of the defense-related events. Proteins from this family were only founded in filamentous fungi previously (56). Furthermore, the phylogenetic analysis revealed two major lineages that significantly distanced from each other. The results suggested that the FIPs could be divided into two groups. At present, only three immunomodulator proteins with cerato-platanin conserved domain have been reported, more evidence or homologs need to be characterized in this new protein family. In this paper, FIP-mco possessed the conserved cerato-platanin domain. It is a new member of the family. The inspiring discovery of FIP-mco means a great progress in the study of the young protein family branch in FIPs. In *M. conica* SH genome, BLAST results suggested the absent of conventional FIP homolog, however, a candidate (scaffold3t326) which shows significant identity (>40%) to ACA and YZP were chosen for analysis of heterologous expression and biological activity.

The crystal structure of FIP-fve has been reported previously (57), and based on the reported protein structure, several homologous FIPs from other origins have predicted their own protein structures. However, based on the sequence alignment results, FIP-mco was homologous only to two reported FIPs (ACA and YZP), whose crystallization structure have not been reported till now. Therefore, it seems impossible to predict the crystal structure of FIP-mco based on available data. Accordingly, structure determination and analysis of FIP-mco will be a new direction for us in subsequent work.

The FIP-mco encoding gene was synthesized, cloned into yeast pPICZαA vector, and was successfully expressed in *P. pastoris* X33. SDS-PAGE revealed a monomer form of the recombinant protein, whose size conformed to the calculated molecular weight of 13.57 kDa. FIPs from the Fve family tend to polymerize in the N-terminal α-helices and form as dimer in solution, therefore, both monomer and dimer form can be observed during expression (15, 42).

In the study of biological activity of rFIP-mco, it can significantly inhibit the proliferation of A549 and HepG2 cancer cells at all the set gradient concentrations. The protein rFIP-mco can remarkably inhibit the proliferation of HepG2 cells at the concentration of 5 μg/ml, which is a relative low effecting concentration reported to date. The effect of rFIP-mco was especially apparent in HepG2 cells, which was similar to that of some FIPs such as rFIP-nha (33). In the determination of the effect on cell viability of the THP1 cells by rFIP-mco at different concentrations, it was found that cell viability of the THP-1 cell was almost not affected. About the different effects of rFIP-mco on A549, HepG2, and the THP-1 cells, the possible cause may be that THP-1 belongs to a kind of blood malignancy while A549 and HepG2 belong to solid tumor. The functional targets for rFIP-mco may be different in different cancer cells, or may express higher in A549 and HepG2 cancer cells while expressing lower or with no expression in the THP1 cells. This also conforms to previous report, which demonstrates that the biological activities of FIPs are quite varied despite high sequence and structure conservation (35). It was considered to be associated with the structural differences at the loop DE and loop FG (58). Moreover, structural differences at the N- terminal α-helix region may be also an important factor in cell surface recognition (30, 59). Besides, we also studied the mechanisms underlying the inhibitory effect of rFIP-mco on A549 and HepG2 cancer cells. The results showed that the proliferation of A549 and HepG2 cancer cells may be inhibited through G0/G1 to S-phase arrest and increased apoptosis. According to previous report, rFIP-ppl (rFIP from *Postia placenta*) could effectively induce MGC823 and HepG2 tumor cell apoptosis in a dose dependent manner, and the apoptotic effects were cell specific (35), which was in accordance with the result in this study. As the increase of the concentration of rFIP-mco, the ratio of apoptotic cells also went ascend. At the concentration of 30 μg/ml, the effect on HepG2 apoptotic cells are more significant, which is consistent with the result of cytotoxicity assay. Moreover, rFIP-mco can apparently repress A549 and HepG2 cancer cell migration and invasion, and rFIP-mco showed a stronger effect on the migration and invasion of A549 cells compared with that on HepG2 cells. The results demonstrated potential application prospect of FIP-mco as antitumor therapeutic agent both in cancer cell proliferation, migration and invasion.

In previous reports on FIPs, they exist as dimers in a dumbbell-shaped structure similar to that of the variable region of immunoglobulin heavy chains, but they exhibit diverse activities (15). It is reported that most FIPs can induce the expression of many cytokines. The recombinant ACA (rACA) has previously been demonstrated to have a LPS-mimetic proinflammatory response toward RAW 264.7 macrophages, and can induce TNF-α and IL-1B production within murine peritoneal macrophages (16). FIP-glu can enhance the transcription of IL-2, IL-3, IL-4, IFN-γ, and TNF-α (60). FIP-gts (*Ganoderma tsugae*) can induce cytokine secretion, and FIP-gaps enhances IL-2 and IFN-γ release from mouse lymphocytes (23). However, FIP-gmi from *Ganoderma microsporum* exhibits the effect of down regulation of TNF-α (28). The induction effect of FIPs on some certain cytokines suggests that they are immune stimulants and can strengthen the immune response of its host, while the down regulation effect suggests they can mitigate inflammatory response.

In this paper, rFIP-mco can significantly reduce the expression levels of cytokines TNF-α, IL-1β, and IL-6. On the effect of TNF-α, rFIP-mco exhibits reduction activity, which is similar to the effect of FIP-gmi on TNF-α, suggesting rFIP-mco can inhibit inflammatory response. Diverse bioactivities of FIPs from different origins may indicate that they have different immunomodulatory mechanisms while binding to the cytokines. ACA from *Auricularia polytricha* enhances the production of TNF-α by LPS-induced RAW 264.7 macrophages (16). YZP can induce the production of IL-6 and IL-10 in B cells (39). Although FIP-mco, ACA and YZP belong to a new branch of the FIP family, the inflammatory activities they displayed were different. According to the reports (27, 61, 62), different responses to FIPs may be associated with small changes in protein structure and folding, to the formation of homo-, di-, tri-, and tetra-mers of rFIPs, or might be relevant to the post-translational modification, all of which can possibly affect receptor binding. As previously referred, different biological activities to cancer cells by FIPs may be related to the structural differences at the loop DE and loop FG. Significant conformation changes were observed between FIP-fve and LZ-8 at the two loop regions of the fibronectin type ΙΙΙ (FNΙΙΙ) domain which may be potential active sites, while the loops of FNΙΙΙ-containing proteins are also usually involved in the recognition of cytokine receptors (59). To elucidate the divergence, it is essential to determine the crystal structure of FIP-mco which is already underway now.

Molecular mechanisms of FIPs on modulating the expression levels of inflammatory cytokines have been reported rarely. It has been previously reported that the expression of TNF-α was closely related with the activation of NF-κB pathway in macrophage cells (63). In order to make clear how the expression levels of cytokines are modulated, the NF-κB signaling pathway was studied in this paper. The results demonstrated that rFIP-mco reduced the expression levels of inflammatory cytokines by suppression of NF-κB signaling pathway. The regulating mechanism is similar to the report of Fip-lti1 and Fip-lti2 (64), in which the anti-inflammmatory properties of Fip-lti1 and Fip-lti2 were also mediated through regulating the NF-κB pathway. In the biological activity study of ACA, ACA directly activates the NF-κB signaling pathway in murine macrophages (16). Combined with the above different cytokine expression levels enhanced or reduced by different FIPs, it indicated that FIPs from different origins may activate or suppress the NF-κB pathway. When FIPs have the function of activating the NF-κB signaling pathway, the expression levels of some cytokines may be induced. While FIPs have the function of suppressing the NF-κB signaling pathway, the expression levels of some cytokines may be reduced. To illustrate definite and clear regulating molecular mechanisms of FIPs on this aspect, the expression levels of more cytokines need to be studied, and more FIPs need to be characterized in this family. As for the reduction effect on inflammatory cytokines by rFIP-mco, it showed that rFIP-mco could mitigate inflammatory response and possessed significant anti-inflammatory effect. The character endowed FIP-mco the prospect of anti-inflammatory medicine development.

As reported in FIP-nha and LZ-9, it is concluded that each FIP has its own unique bioactivity profile (27). In our study, it also demonstrated this point. They are diversified in biological activity and possess independent features, behind which the possible cause in structure differences is very interesting. For subsequent work, further study on rFIP-mco using animal models is underway. Further function mechanism of FIPs on cancer cells and cytokines is also an interesting aspect and deserves deep study on crystal structure. As a new member of the branch family of FIPs, the found and biological activity determination of FIP-mco from the precious rare edible mushroom *M. conica* SH has enormous research and application value. It will promote the study of the protein divergence in the FIP family, the molecular mechanism of FIPs on cytokines and cancer cells. FIP-mco possesses wide application prospect as antitumor adjuvant in medical and pharmaceutical area, and also in health care and food industry.

## Data Availability Statement

All datasets presented in this study are included in the article/**Supplementary Material**.

## Author Contributions

GW and YS carried out the study and drafted the manuscript. LS and TD were responsible for immunology experiment. PL and HZ contributed to gene cloning and expression. XT conceived the experimental design and guided the study. All authors contributed to the article and approved the submitted version.

## Funding

The project was supported by the funding of Shanghai Agricultural Science Committee Major Project 2020 (2–1), Shanghai Agriculture Applied Technology Development Program (Z20180103), Shanghai Agricultural Science Committee Youth Talents Development Plan No. 2018 (1–32) and 2018 (1–34), SAAS Program for Excellent Research Team No. 2017 (B-07), Science and Technology Support Program KJZC202008, the National Natural Science Foundation of China (31801511 and 31100055).

## Conflict of Interest

The authors declare that the research was conducted in the absence of any commercial or financial relationships that could be construed as a potential conflict of interest.

## Abbreviations

BMGY, buffered glycerol-complex medium; CCK-8, Cell-Counting Kit 8; FIP, fungal immunomodulatory protein; FIP-nha, FIP from *Nectria haematococca;* FIP-ppl, *Postia placenta;* JTT, Jones-Taylor-Thornton; NJ, Neighbor-Joining; OD, optical density; SDS-PAGE, sodium dodecyl sulfate-polyacrylamide gel electrophoresis; YNB, yeast nitrogen base; YPD, yeast extract peptone dextrose; FIP-mco, FIP from *M. conica* SH; rFIP-mco, the recombinant protein of FIP-mco.

## References

[1] Kalac P A review of chemical composition and nutritional value of wild-growing and cultivated mushrooms. J Sci Food Agric (2013) 93:209–18. 10.1002/jsfa.5960 23172575

[2] Xu N Lu Y Hou J Liu C Sun Y A polysaccharide purified from *Morchella conica Pers*. Prevents oxidative stress induced by H_2_O_2_ in human embryonic kidney (HEK) 293T cells. Int J Mol Sci (2018) 19:4027. 10.3390/ijms19124027 PMC6320779 30551572

[3] Wasser SP Current findings, future trends, and unsolved problems in studies of medicinal mushrooms. Appl Microbiol Biotechnol (2011) 89:1323–32. 10.1007/s00253-010-3067-4 21190105

[4] Pfab R Haberl B Kleber J Zilker T Cerebellar effects after consumption of edible morels (*Morchella conica*, *Morchella esculenta*). Clin Toxicol (Phila) (2008) 46:259–60. 10.1080/15563650701206715 18344109

[5] Vieira V Fernandes A Barros L Glamoclija J Ciric A Stojkovic D Wild *Morchella conica* Pers. from different origins: a comparative study of nutritional and bioactive properties. J Sci Food Agric (2016) 96:90–8. 10.1002/jsfa.7063 25546397

[6] Li QZ Wang XF Zhou XW Recent status and prospects of the fungal immunomodulatory protein family. Crit Rev Biotechnol (2011) 31:365–75. 10.3109/07388551.2010.543967 21651437

[7] Meng F Zhou B Lin R Jia L Liu X Deng P Extraction optimization and in vivo antioxidant activities of exopolysaccharide by *Morchella esculenta* SO-01. Bioresour Technol (2010) 101:4564–9. 10.1016/j.biortech.2010.01.113 20153962

[8] Wei W Luo X Zheng L Yu M Jiang N Xu XY Isolation of a wild Morchella spp. strain and the effects of its extract on ethanol-induced gastric mucosal lesions in rats. Z Naturforsch C J Biosci (2011) 66:55–62. 10.5560/znc.2011.66c0055 21476437

[9] Tietel Z Masaphy S True morels (Morchella)-nutritional and phytochemical composition, health benefits and flavor: a review. Crit Rev Food Sci Nutr (2018) 58:1888–901. 10.1080/10408398.2017.1285269 28350213

[10] Fu L Wang Y Wang J Yang Y Hao L Evaluation of the antioxidant activity of extracellular polysaccharides from *Morchella esculenta*. Food Funct (2013) 4:871–9. 10.1039/c3fo60033e 23598461

[11] Elmastas M Turkekul I Ozturk L Gulcin I Isildak O Aboul-Enein HY Antioxidant activity of two wild edible mushrooms (*Morchella vulgaris* and *Morchella esculanta*) from North Turkey. Comb Chem High Throughput Screen (2006) 9:443–8. 10.2174/138620706777698544 16842225

[12] Kuo M Dewsbury DR O'donnell K Carter MC Rehner SA Moore JD Taxonomic revision of true morels (Morchella) in Canada and the United States. Mycologia (2012) 104:1159–77. 10.3852/11-375 22495449

[13] Winder RS Cultural studies of *Morchella elata*. Mycol Res (2006) 110:612–623. 10.1016/j.mycres.2006.02.003 16769512

[14] Huang M Zhang S Zhang M Ou S Pan Z Effects of polysaccharides from *Morchella conica* on nitric oxide production in lipopolysaccharide-treated macrophages. Appl Microbiol Biotechnol (2012) 94:763–71. 10.1007/s00253-011-3711-7 22159604

[15] El Enshasy HA Hatti-Kaul R Mushroom immunomodulators: unique molecules with unlimited applications. Trends Biotechnol (2013) 31:668–77. 10.1016/j.tibtech.2013.09.003 24125745

[16] Sheu F Chien PJ Hsieh KY Chin KL Huang WT Tsao CY Purification, cloning, and functional characterization of a novel immunomodulatory protein from *Antrodia camphorata* (bitter mushroom) that exhibits TLR2-dependent NF-κB activation and M1 polarization within murine macrophages. J Agric Food Chem (2009) 57:4130–41. 10.1021/jf900469a 19371137

[17] Sheu F Chien P Chien A Chen Y Chin K Isolation and characterization of an immunomodulatory protein (APP) from the Jew’s Ear mushroom *Auricularia polytricha*. Food Chem (2004) 87:593–600. 10.1016/j.foodchem.2004.01.015

[18] Wang Y Gao YN Bai R Chen HY Wu YY Shang JJ Identification of a novel anti-cancer protein, FIP-bbo, from *Botryobasidium botryosum* and protein structure analysis using molecular dynamic simulation. Sci Rep (2019) 9:5818. 10.1038/s41598-019-42104-1 30967569 PMC6456589

[19] Lin J-W Guan S-Y Duan Z-W Shen Y-H Fan W-L Chen L-J Gene cloning of a novel fungal immunomodulatory protein from *Chroogomphis rutilus* and its expression in *Pichia pastoris*. J Chem Technol Biotechnol (2016) 91:2761–8. 10.1002/jctb.4881

[20] Li S Jiang Z Sun L Liu X Huang Y Wang F Characterization of a new fungal immunomodulatory protein, FIP-dsq2 from *Dichomitus squalens*. J Biotechnol (2017) 246:45–51. 10.1016/j.jbiotec.2017.02.006 28202377

[21] Ko JL Hsu CI Lin RH Kao CL Lin JY A new fungal immunomodulatory protein, FIP-fve isolated from the edible mushroom, *Flammulina velutipes* and its complete amino acid sequence. Eur J Biochem (1995) 228:244–9. 10.1111/j.1432-1033.1995.tb20256.x 7705335

[22] Lin J Zhong M Chen L Li H Ma H Guo Z FIP-gap. (2011). Available at: https://www.uniprot.org/uniprot/G5CJT8 [Accessed December 14, 2011].

[23] Zhou S Guan S Duan Z Han X Zhang X Fan W Molecular cloning, codon-optimized gene expression, and bioactivity assessment of two novel fungal immunomodulatory proteins from *Ganoderma applanatum* in Pichia. Appl Microbiol Biotechnol (2018) 102:5483–94. 10.1007/s00253-018-9022-5 29705959

[24] González Muñoz A Botero Orozco KJ López Gartner GA Finding of a novel fungal immunomodulatory protein coding sequence in *Ganoderma australe*. Rev Colombiana Biotecnol (2014) 16:90–5. 10.15446/rev.colomb.biote.v16n2.38747

[25] Xu H Kong YY Chen X Guo MY Bai XH Lu YJ Recombinant FIP-gat, a fungal immunomodulatory protein from ganoderma atrum, induces growth inhibition and cell death in breast cancer cells. J Agric Food Chem (2016) 64:2690–8. 10.1021/acs.jafc.6b00539 26996414

[26] Kino K Yamashita A Yamaoka K Watanabe J Tanaka S Ko K Isolation and characterization of a new immunomodulatory protein, Ling Zhi-8 (LZ-8), from *Ganoderma lucidium*. J Biol Chem (1989) 264:472–8. 2909532

[27] Bastiaan-Net S Chanput W Hertz A Zwittink RD Mes JJ Wichers HJ Biochemical and functional characterization of recombinant fungal immunomodulatory proteins (rFIPs). Int Immunopharmacol (2013) 15:167–75. 10.1016/j.intimp.2012.11.003 23174509

[28] Lin C-H Sheu G-T Lin Y-W Yeh C-S Huang Y-H Lai Y-C A new immunomodulatory protein from *Ganoderma microsporum* inhibits epidermal growth factor mediated migration and invasion in A549 lung cancer cells. Process Biochem (2010) 45:1537–42. 10.1016/j.procbio.2010.06.006

[29] Zhou X Xie M Hong F Li Q Lin J Genomic cloning and characterization of a FIP-gsi gene encoding a fungal immunomodulatory protein from *Ganoderma sinense* Zhao et al. (Aphyllophoromycetideae). Int J Med Mushrooms (2009) 11:77–86. 10.1615/IntJMedMushr.v11.i1.90

[30] Lin W-H Hung C-H Hsu C-I Lin J-Y Dimerization of the N-terminal amphipathic α-helix domain of the fungal immunomodulatory protein from *Ganoderma tsugae* (Fip-gts) defined by a yeast two-hybrid system and site-directed mutagenesis. J Biol Chem (1997) 272:20044–8. 10.1074/jbc.272.32.20044 9242675

[31] Diling C Chaoqun Z Jian Y Jian L Jiya NS Yizhen X Immunomodulatory activities of a fungal protein extracted from *Hericium erinaceus* through regulating the gut Microbiota. Front Immunol (2017) 8:666. 10.3389/fimmu.2017.00666 28713364 PMC5492111

[32] Pushparajah V Fatima A Chong CH Gambule TZ Chan CJ Ng ST Characterisation of a new fungal immunomodulatory protein from Tiger milk mushroom, *Lignosus rhinocerotis*. Sci Rep (2016) 6:30010. 10.1038/srep30010 27460640 PMC4962085

[33] Li S Nie Y Ding Y Shi L Tang X Recombinant expression of a novel fungal immunomodulatory protein with human tumor cell antiproliferative activity from *Nectria haematococca*. Int J Mol Sci (2014) 15:17751–64. 10.3390/ijms151017751 PMC4227187 25272229

[34] Chang HH Yeh CH Sheu F A novel immunomodulatory protein from *Poria cocos* induces Toll-like receptor 4-dependent activation within mouse peritoneal macrophages. J Agric Food Chem (2009) 57:6129–39. 10.1021/jf9011399 19548679

[35] Li SY Shi LJ Ding Y Nie Y Tang XM Identification and functional characterization of a novel fungal immunomodulatory protein from *Postia placenta*. Food Chem Toxicol (2015) 78:64–70. 10.1016/j.fct.2015.01.013 25662032

[36] Li S Zhao L Xu W Jiang Z Kang J Wang F Identification and characterisation of a novel protein FIP-sch3 from *Stachybotrys chartarum*. PloS One (2016) 11:e0168436. 10.1371/journal.pone.0168436 27997578 PMC5173029

[37] Li S Jiang Z Xu W Xie Y Zhao L Tang X FIP-sch2, a new fungal immunomodulatory protein from *Stachybotrys chlorohalonata*, suppresses proliferation and migration in lung cancer cells. Appl Microbiol Biotechnol (2017) 101:3227–35. 10.1007/s00253-016-8030-6 28078399

[38] Li F Wen H Liu X Zhou F Chen G Gene cloning and recombinant expression of a novel fungal immunomodulatory protein from *Trametes versicolor*. Protein Expr Purif (2012) 82:339–44. 10.1016/j.pep.2012.01.015 22342678

[39] Kuan YC Wu YJ Hung CL Sheu F *Trametes versicolor* protein YZP activates regulatory B lymphocytes - gene identification through de novo assembly and function analysis in a murine acute colitis model. PloS One (2013) 8:e72422. 10.1371/journal.pone.0072422 24019869 PMC3760908

[40] Hsu HC Hsu CI Lin RH Kao CL Lin JY Fip-vvo, a new fungal immunomodulatory protein isolated from *Volvariella volvacea*. Biochem J (1997) 323(Pt 2):557–65. 10.1042/bj3230557 PMC1218355 9163352

[41] Wang Y Wāng Y Gao Y Li Y Wan JN Yang RH Discovery and characterization of the highly active fungal immunomodulatory protein Fip-vvo82. J Chem Inf Model (2016) 56:2103–14. 10.1021/acs.jcim.6b00087 27649295

[42] Li QZ Zheng YZ Zhou XW Fungal immunomodulatory proteins: characteristic, potential antitumor activities and their molecular mechanisms. Drug Discovery Today (2019) 24:307–14. 10.1016/j.drudis.2018.09.014 30266655

[43] Liao CH Hsiao YM Lin CH Yeh CS Wang JC Ni CH Induction of premature senescence in human lung cancer by fungal immunomodulatory protein from *Ganoderma tsugae*. Food Chem Toxicol (2008) 46:1851–9. 10.1016/j.fct.2008.01.044 18329152

[44] Chiu LY Hu ME Yang TY Hsin IL Ko JL Tsai KJ Immunomodulatory protein from *Ganoderma microsporum* induces pro-death autophagy through Akt-mTOR-p70S6K Pathway inhibition in multidrug resistant lung cancer cells. PloS One (2015) 10:e0125774. 10.1371/journal.pone.0125774 25946033 PMC4422711

[45] Morin E Kohler A Baker AR Foulongne-Oriol M Lombard V Nagye LG Genome sequence of the button mushroom *Agaricus bisporus* reveals mechanisms governing adaptation to a humic-rich ecological niche. Proc Natl Acad Sci U S A (2012) 109:17501–6. 10.1073/pnas.1206847109 PMC3491501 23045686

[46] Chen L Gong Y Cai Y Liu W Zhou Y Xiao Y Genome sequence of the edible cultivated mushroom *Lentinula edodes* (Shiitake) reveals insights into lignocellulose degradation. PloS One (2016) 11:e0160336. 10.1371/journal.pone.0160336 27500531 PMC4976891

[47] Wu B Xu Z Knudson A Carlson A Chen N Kovaka S Genomics and development of *Lentinus tigrinus*: a white-rot wood-decaying mushroom with dimorphic fruiting bodies. Genome Biol Evol (2018) 10:3250–61. 10.1093/gbe/evy246 PMC6305247 30398645

[48] Murat C Payen T Noel B Kuo A Morin E Chen J Pezizomycetes genomes reveal the molecular basis of ectomycorrhizal truffle lifestyle. Nat Ecol Evol (2018) 2:1956–65. 10.1038/s41559-018-0710-4 30420746

[49] Stajich JE Wilke SK Ahrén D Au CH Birren BW Borodovsky M Insights into evolution of multicellular fungi from the assembled chromosomes of the mushroom *Coprinopsis cinerea* (*Coprinus cinereus*). Proc Natl Acad Sci U S A (2010) 107:11889–94. 10.1073/pnas.1003391107 PMC2900686 20547848

[50] Riley R Salamov AA Brown DW Nagy LG Floudas D Held BW Extensive sampling of basidiomycete genomes demonstrates inadequacy of the white-rot/brown-rot paradigm for wood decay fungi. Proc Natl Acad Sci U S A (2014) 111:9923–8. 10.1073/pnas.1400592111 PMC4103376 24958869

[51] Edgar RC MUSCLE: multiple sequence alignment with high accuracy and high throughput. Nucleic Acids Res (2004) 32:1792–7. 10.1093/nar/gkh340 PMC390337 15034147

[52] Buchan DWA Minneci F Nugent TCO Bryson K Jones DT Scalable web services for the PSIPRED Protein Analysis Workbench. Nucleic Acids Res (2013) 41:349–57. 10.1093/nar/gkt381 PMC3692098 23748958

[53] Kumar S Stecher G Tamura K MEGA7: molecular evolutionary genetics analysis version 7.0 for bigger datasets. Mol Biol Evol (2016) 33:1870–4. 10.1093/molbev/msw054 PMC8210823 27004904

[54] Gill SC Von Hippel PH Calculation of protein extinction coefficients from amino acid sequence data. Anal Biochem (1989) 182:319–26. 10.1016/0003-2697(89)90602-7 2610349

[55] Zhou S-G Zhou S-F Huang H-Q Chen J-W Huang M Liu P-Q Proteomic analysis of hypertrophied myocardial protein patterns in renovascularly hyper-tensive and spontaneously hypertensive rats. J Proteome Res (2006) 5:2901–8. 10.1021/pr050456l 17081041

[56] Gaderer R Bonazza K Seidl-Seiboth V Cerato-platanins: a fungal protein family with intriguing properties and application potential. Appl Microbiol Biotechnol (2014) 98:4795–803. 10.1007/s00253-014-5690-y PMC4024134 24687753

[57] Paaventhan P Joseph JS Seow SV Vaday S Robinson H Chua KY A 1.7Å structure of fve, a member of the new fungal immunomodulatory protein family. J Mol Biol (2003) 332:461–70. 10.1016/s0022-2836(03)00923-9 12948495

[58] Huang L Sun F Liang C He YX Bao R Liu L Crystal structure of LZ-8 from the medicinal fungus *Ganoderma lucidium*. Proteins (2009) 75:524–7. 10.1002/prot.22346 19137616

[59] Williams AF Barclay AN The immunoglobulin superfamily–domains for cell surface recognition. Annu Rev Immunol (1988) 6:381–405. 10.1146/annurev.iy.06.040188.002121 3289571

[60] Li QZ Wang XF Bao TW Ran L Juan L Zhou XW *In vitro* synthesis of a recombinant fungal immunomodulatory protein from Lingzhi or Reishi medicinal mushroom, *Ganoderma lucidum* (W. Curt.: Fr.) P. Karst. (Aphyllophoromycetideae) and analysis of its immunomodulatory activity. Int J Med Mushrooms (2010) 12:347–58. 10.1615/IntJMedMushr.v12.i4.20

[61] Ou C-C Hsiao Y-M Wang W-H Ko J-L Lin M-Y Stability of fungal immunomodulatory protein, FIP-gts and FIP-fve, in IFN-γ production. Food Agric Immunol (2009) 20:319–32. 10.1080/09540100903247688

[62] Lin WH Hung CH Hsu CI Lin JY Dimerization of the N-terminal amphipathic alpha-helix domain of the fungal immunomodulatory protein from Ganoderma tsugae (Fip-gts) defined by a yeast two-hybrid system and site-directed mutagenesis. J Biol Chem (1997) 272:20044–8. 10.1074/jbc.272.32.20044 9242675

[63] Li H Lin X Positive and negative signaling components involved in TNF-α-induced NF-κB activation. Cytokine (2008) 41:1–8. 10.1016/j.cyto.2007.09.016 18068998

[64] Gao Y Wáng Y Wāng Y Wu Y Chen H Yang R Function of novel fungal immunomodulatory proteins Fip-lti1 and Fip-lti2 from *Lentinus tigrinus* in Concanavalin A-induced liver oxidative injury. Oxid Med Cell Longev (2019) 2019:3139689. 10.1155/2019/3139689 31198490 PMC6526528

