# Supplementary material for: Identification and Functional Characterization of a Novel Immunomodulatory Protein From Morchella conica SH
Source: Front Immunol. 2020 Oct 26;11:559770. doi: 10.3389/fimmu.2020.559770 (PMC7649207; doi:10.3389/fimmu.2020.559770)
Supplement: Supplementary file 1 [file DataSheet_1.docx]

**Supplementary Information**

**Table S1. Summary Table of the Reported Fungal Immunomodulatory Proteins (FIPs)**

| species | FIPs | AA length | accession No. | ref. |
| --- | --- | --- | --- | --- |
| *Antrodia camphorata* | ACA | 137 | AY56969.1 | (1) |
| *Auricularia polytricha* | APP | - | - | (2) |
| *Botryobasidium botryosum* | FIP-bbo | 112 | KDQ10166.1 | (3) |
| *Chroogomphus rutilus* | FIP-cru | 113 | KT210088.1 | (4) |
| *Dichomitus squalens* | FIP-dsq2 | 111 | XP 007363541.1 | (5) |
| *Flammulina velutipes* | FIP-fve | 114 | GU388420.1 | (6) |
| *Ganoderma applanatum* | FIP-gap1 | 113 | JN167598.1 | (7) |
| *Ganoderma applanatum* | FIP-gap2 | 113 | ART88472.1 | (8) |
| *Ganoderma australe* | c13717 | 137 |  | (9) |
| *Ganoderma atrum* | FIP-gat | 111 | - | (10) |
| *Ganoderma japonicum* | FIP-gja | 111 | AAX98241 |  |
| *Ganoderma lucidum* | FIP-glu (LZ-8) | 112 | M58032.1 | (11) |
| *Ganoderma lucidum* | FIP-glu2 (LZ-9) | 111 |  | (12) |
| *Ganoderma microsporum* | FIP-GMI | 111 | - | (13) |
| *Ganoderma sinense* | FIP-gsi | 111 | AY449805.1 | (14) |
| *Ganoderma tsugae* | FIP-gts | 110 |  | (15) |
| *Hericium erinaceus* | FIP-her |  | - | (16) |
| *Lignosus rhinocerotis* | FIP-lrh | 112 | - | (17) |
| *Nectria haematococca* | FIP-nha | 113 | GG698920.1 | (18) |
| *Poria cocos* | PCP | - | JN571084.1 | (19) |
| *Postia placenta* | FIP-ppl | 125 | KJ818121.1 | (20) |
| *Stachybotrys chartarum* | FIP-sch3 | 112 | KFA81641.1 | (21) |
| *Stachybotrys chlorohalonata* | FIP-sch2 | 112 | AQQ80204.1 | (22) |
| *Trametes versicolor* | FIP-tvc | 111 | - | (23) |
| *Trametes versicolor* | YZP | 119 | KC297708.1 | (24) |
| *Volvariella volvacea* | FIP-vvo | 113 | - | (25) |
| *V. volvacea* | FIP-vvo82 | 113 |  | (26) |

1. Sheu F, et al. Purification, cloning, and functional characterization of a novel immunomodulatory protein from *Antrodia camphorata* (bitter mushroom) that exhibits TLR2-dependent NF-κB activation and M1 polarization within murine macrophages. *J Agric Food Chem*. (2009) 57**:**4130–4141. doi: 10.1021/jf900469a

2. Sheu F, Chien P, Chien A, Chen Y, Chin K. Isolation and characterization of an immunomodulatory protein (APP) from the Jew's Ear mushroom *Auricularia polytricha*. *Food Chem*. (2004) 87**:**593–600. doi: 10.1016/j.foodchem.2004.01.015

3. Wang Y, et al. Identification of a novel anti-cancer protein, FIP-bbo, from *Botryobasidium botryosum* and protein structure analysis using molecular dynamic simulation. *Sci Rep*. (2019) 9**:**5818. doi: 10.1038/s41598-019-42104-1

4. Lin J-W, et al. Gene cloning of a novel fungal immunomodulatory protein from *Chroogomphis rutilus* and its expression in *Pichia pastoris*. *J Chem Technol Biotechnol*. (2016) 91**:**2761–2768. doi: 10.1002/jctb.4881

5. Li S, et al. Characterization of a new fungal immunomodulatory protein, FIP-dsq2 from *Dichomitus squalens*. *J Biotechnol*. (2017) 246**:**45–51. doi: 10.1016/j.jbiotec.2017.02.006

6. Ko JL, Hsu CI, Lin RH, Kao CL, Lin JY. A new fungal immunomodulatory protein, FIP-fve isolated from the edible mushroom, *Flammulina velutipes* and its complete amino acid sequence. *Eur J Biochem*. (1995) 228**:**244–249. doi:

7. Lin J, et al. Molecular cloning of a fungal immunomodulatory protein gene, FIP-gap, from *Ganoderma applanatum*. *UniProtKB - G5CJT8 (G5CJT8_9APHY)*. (2011). doi:

8. Zhou S, et al. Molecular cloning, codon-optimized gene expression, and bioactivity assessment of two novel fungal immunomodulatory proteins from *Ganoderma applanatum* in Pichia. *Appl Microbiol Biotechnol*. (2018) 102**:**5483–5494. doi: 10.1007/s00253-018-9022-5

9. González Muñoz A, Botero Orozco KJ, López Gartner GA. Finding of a novel fungal immunomodulatory protein coding sequence in *Ganoderma australe*. *Rev Colombiana Biotecnol*. (2014) 16**:**90–95. doi: 10.15446/rev.colomb.biote.v16n2.38747

10. Xu H, et al. Recombinant FIP-gat, a fungal immunomodulatory protein from ganoderma atrum, induces growth inhibition and cell death in breast cancer cells. *J Agric Food Chem*. (2016) 64**:**2690–2698. doi: 10.1021/acs.jafc.6b00539

11. Kino K, et al. Isolation and characterization of a new immunomodulatory protein, Ling Zhi-8 (LZ-8), from *Ganoderma lucidium*. *J Biol Chem*. (1989) 264**:**472–478. doi:

12. Bastiaan-Net S, et al. Biochemical and functional characterization of recombinant fungal immunomodulatory proteins (rFIPs). *Int Immunopharmacol*. (2013) 15**:**167–175. doi: 10.1016/j.intimp.2012.11.003

13. Lin C-H, et al. A new immunomodulatory protein from *Ganoderma microsporum* inhibits epidermal growth factor mediated migration and invasion in A549 lung cancer cells. *Process Biochem*. (2010) 45**:**1537–1542. doi: 10.1016/j.procbio.2010.06.006

14. Zhou X, Xie M, Hong F, Li Q, Lin J. Genomic cloning and characterization of a FIP-gsi gene encoding a fungal immunomodulatory protein from *Ganoderma sinense* Zhao et al. (Aphyllophoromycetideae). *Int J Med Mushrooms*. (2009) 11**:**77–86. doi: 10.1615/IntJMedMushr.v11.i1.90

15. Lin W-H, Hung C-H, Hsu C-I, Lin J-Y. Dimerization of the N-terminal amphipathic α-helix domain of the fungal immunomodulatory protein from *Ganoderma tsugae* (Fip-gts) defined by a yeast two-hybrid system and site-directed mutagenesis. *J Biol Chem*. (1997) 272**:**20044–20048. doi: 10.1074/jbc.272.32.20044

16. Diling C, et al. Immunomodulatory activities of a fungal protein extracted from *Hericium erinaceus* through regulating the gut Microbiota. *Front Immunol*. (2017) 8**:**666. doi: 10.3389/fimmu.2017.00666

17. Pushparajah V, et al. Characterisation of a new fungal immunomodulatory protein from Tiger milk mushroom, *Lignosus rhinocerotis*. *Sci Rep*. (2016) 6**:**30010. doi: 10.1038/srep30010

18. Li S, Nie Y, Ding Y, Shi L, Tang X. Recombinant expression of a novel fungal immunomodulatory protein with human tumor cell antiproliferative activity from *Nectria haematococca*. *Int J Mol Sci*. (2014) 15**:**17751–17764. doi: 10.3390/ijms151017751

19. Chang HH, Yeh CH, Sheu F. A novel immunomodulatory protein from *Poria cocos* induces Toll-like receptor 4-dependent activation within mouse peritoneal macrophages. *J Agric Food Chem*. (2009) 57**:**6129–6139. doi: 10.1021/jf9011399

20. Li SY, Shi LJ, Ding Y, Nie Y, Tang XM. Identification and functional characterization of a novel fungal immunomodulatory protein from *Postia placenta*. *Food Chem Toxicol*. (2015) 78**:**64–70. doi: 10.1016/j.fct.2015.01.013

21. Li S, et al. Identification and characterisation of a novel protein FIP-sch3 from *Stachybotrys chartarum*. *PLoS One*. (2016) 11**:**e0168436. doi: 10.1371/journal.pone.0168436

22. Li S, et al. FIP-sch2, a new fungal immunomodulatory protein from *Stachybotrys chlorohalonata*, suppresses proliferation and migration in lung cancer cells. *Appl Microbiol Biotechnol*. (2017) 101**:**3227–3235. doi: 10.1007/s00253-016-8030-6

23. Li F, Wen H, Liu X, Zhou F, Chen G. Gene cloning and recombinant expression of a novel fungal immunomodulatory protein from *Trametes versicolor*. *Protein Expr Purif*. (2012) 82**:**339–344. doi: 10.1016/j.pep.2012.01.015

24. Kuan YC, Wu YJ, Hung CL, Sheu F. *Trametes versicolor* protein YZP activates regulatory B lymphocytes - gene identification through de novo assembly and function analysis in a murine acute colitis model. *PLoS One*. (2013) 8**:**e72422. doi: 10.1371/journal.pone.0072422

25. Hsu HC, Hsu CI, Lin RH, Kao CL, Lin JY. Fip-vvo, a new fungal immunomodulatory protein isolated from *Volvariella volvacea*. *Biochem J*. (1997) 323 (Pt 2)**:**557–565. doi: 10.1042/bj3230557

26. Wang Y, et al. Discovery and characterization of the highly active fungal immunomodulatory protein Fip-vvo82. *J Chem Inf Model*. (2016) 56**:**2103–2114. doi: 10.1021/acs.jcim.6b00087
